# Impact of Sarcopenic Diabetes on Outcomes and Mortality in Older Adults Hospitalized for Hip Fracture: A Nested Case–Control Study Within a Real-World Evidence Cohort

**DOI:** 10.3390/nu17162616

**Published:** 2025-08-12

**Authors:** Elisa García-Tercero, Daniela Villalon Rubio, Ángel Belenguer-Varea, Cristina Cunha-Pérez, José Viña, Francisco José Tarazona-Santabalbina

**Affiliations:** 1 Geriatric Medicine Department, Hospital Universitario de la Ribera, 46600 Alzira, Spain; 2 School of Doctorate, Universidad Católica de Valencia San Vicente Mártir, 46001 Valencia, Spain; 3 Centro de Investigación Biomédica en Red Fragilidad y Envejecimiento Saludable (CIBERFES), 28029 Madrid, Spain; 4 Departament of Physiology, Universitat de Valencia, 46010 Valencia, Spain; 5 Medical School, Universidad Católica de Valencia San Vicente Mártir, 46001 Valencia, Spain

**Keywords:** sarcopenic diabetes, hip fractures, older adults, mortality, nutritional status, frailty, in-hospital outcomes, sarcopenia screening

## Abstract

Background: This study addresses the prevalence and outcomes of sarcopenic diabetes among older adults hospitalized for hip fractures, highlighting its association with nutritional status, frailty, in-hospital outcomes, and mortality. With the global incidence of hip fractures anticipated to rise significantly, understanding these associations is crucial, especially considering the higher prevalence of sarcopenia in diabetic patients, which exacerbates outcomes. Methods: An observational, unicentric case–control study nested within a real-world data cohort was conducted. It included 2631 older adults (aged ≥ 70 years) hospitalized for hip fractures at the Hospital Universitario de la Ribera, Spain, from 2014 to 2021. Diabetic patients were classified as cases, and non-diabetic patients served as controls. The study examined demographic variables, comorbidities, nutritional status, geriatric syndromes, and mortality, using the SARC-F questionnaire for sarcopenia screening. Results: The study found that cases (diabetic patients) presented with higher comorbidities, longer hospital stays, and a higher prevalence of sarcopenia, significantly impacting in-hospital and long-term mortality rates. The prevalence of possible sarcopenia was notably higher at 61.5% among diabetic patients. Sarcopenia was strongly correlated with worse functional outcomes and higher mortality. Conclusions: Sarcopenic diabetes significantly increases the risk of adverse outcomes and mortality in older adults with hip fractures. The findings underscore the importance of routine screening for diabetes and sarcopenia in this patient population to mitigate risks and improve rehabilitation outcomes.

## 1. Introduction

Osteoporotic hip fracture is a major health problem in the elderly, and the annual incidence worldwide is expected to increase to over 6 million by 2050 [1]. Hip fracture is a major health problem due to the high risk of morbidity, severe disability, clinical and socioeconomic burden, and mortality [2]. Mortality is very high, with up to 10% of patients hospitalized with a hip fracture dying in the month after discharge and nearly 30% dying in the first year [3]. Similarly, the associated disability in survivors after discharge dramatically increases the costs, which are derived from the percentage of patients who have serious difficulty walking independently or never regain the ability to walk [4].

Sarcopenia is defined as a disorder of skeletal muscle with accelerated loss of muscle function and muscle mass associated with ageing and is associated with adverse outcomes including frailty, disability, morbidity, and mortality. The prevalence of sarcopenia ranges from 12.9% to 40.4%, while the incidence varies from 1 to 6% in people aged 40–79 years to 3–6% in people aged 85 years and older [5,6]. The prevalence of sarcopenia is high in hip fracture patients, higher in men (37–61%) than in women (11–26%), and correlates well with long-term mortality [6]. In addition, sarcopenia in hip fracture patients delays functional recovery in hip fracture patients undergoing in-hospital rehabilitation [7].

The prevalence of sarcopenia is higher in patients with diabetes, reaching 27.9% of the patients studied [8]. Type 2 diabetes causes damage to human organs and systems, including an increased risk of fractures [9] due to impaired bone quality rather than a decrease in bone mineral density [10]. Similarly, the estimated prevalence of sarcopenia in people with diabetes is 18% [11], higher than in people of the same age and sex without sarcopenia. Diabetes and diabetic osteoporosis are associated with a higher risk of sarcopenia due to a bidirectional influence between the muscle–bone system and the muscle–endocrine system [12]. Type 2 diabetes increases the risk of poor outcomes during hospitalization for hip fracture. Patients with type 2 diabetes have a higher risk of pressure ulcers [13], delayed surgery [14], longer hospital stays, poorer functional status, discharge to higher-level care [15], and higher in-hospital and long-term mortality rates [16].

An increasedhigher risk of sarcopenia among type 2 diabetes patients with hip fracture has been reported [17], but the relationship between sarcopenia, type 2 diabetes, and hip fracture in terms of outcomes including mortality remains unclear. Recently, a proposal for muscle screening in type 2 diabetes has been published, defining the concept of diabetic sarcopenia (DS) [18].

The aim of this study was to analyze the prevalence of type 2 diabetes and diabetic sarcopenia in older adults hospitalized for hip fracture evaluated by this new DS algorithm [18] and its association with nutritional status, prevalence of frailty, in-hospital outcomes, and in-hospital and long-term mortality.

## 2. Materials and Methods

### 2.1. Study Design and Subjects

To realize the objectives of the study, an observational, unicentric, case–control study was designed, nested in a real-world evidence cohort of older adults aged 70 years and older hospitalized for hip fracture. A total of 2631 consecutive patients admitted for hip fracture to the Hospital Universitario de la Ribera (HULR) (Alzira, Valencia, Spain) were recruited between 1 January 2014 and 31 December 2021. HULR is a tertiary care hospital serving a population of 300,205 inhabitants, 13.5% of whom are aged 70 years or older.

All diabetic patients aged 70 years or older hospitalized with hip fracture during the recruitment period were considered as cases, and hip fracture patients without diabetes hospitalized with hip fracture were included in the control group. All patients with pathological fractures and life expectancy of less than six months were excluded.

### 2.2. Sample Size

Data were collected from 2631 (753 diabetic and 1878 non-diabetic) consecutive, non-selected patients admitted to the hospital with hip fracture. The calculated power of the study with the recruited sample, with a fixed alpha error of 5% and a difference in 5-year mortality between diabetic and non-diabetic patients with hip fracture of 5%, was 95.78%.

### 2.3. Outcome Measures

Demographic variables such as age and sex, fracture type, surgical delay, length of stay, comorbidities and the Charlson comorbidity index (CCI) [19], American Society of Anesthesiology physical classification status system (ASA) [20], All Patient Refined Diagnosis Related Groups (APR-DRGs) [21], Controlling Nutritional Status (CONUT) score [22], geriatric syndromes such as delirium, pressure ulcers, and cognitive impairment, red blood cell transfusion, adverse events during hospitalization and in-hospital, short- and long-term mortality were collected. The CONUT (Controlling Nutritional Status) score was calculated using three biochemical parameters, serum albumin concentration (g/dL), total lymphocyte count (/mm^3^), and total cholesterol level (mg/dL), which reflect protein reserves, immune function, and lipid metabolism, respectively. Each parameter was assigned a score according to predefined cut-off values: serum albumin (≥3.50 = 0 points, 3.00–3.49 = 2 points, 2.50–2.99 = 4 points, <2.50 = 6 points), total lymphocyte count (≥1600 = 0 points, 1200–1599 = 1 point, 800–1199 = 2 points, <800 = 3 points), and total cholesterol (≥180 = 0 points, 140–179 = 1 point, 100–139 = 2 points, <100 = 3 points). The final CONUT score was obtained by summing the individual scores of these three parameters, with higher scores indicating a greater degree of undernutrition [22]. The risk of DS in patients with diabetic hip fracture was evaluated by a new algorithm which takes into account the SARC-F values [23] and the presence of one or more specific risk factors like HbA1C ≥ 8%, more than 5 years since T2DM onset, the presence of diabetic chronic complications (like recent major trauma with/without complication or sustained immobilization or reduced mobility due to fracture), and use of blood glucose lowering drugs (sulphonylureas, glinides or SGLt2) [18].

Because all diabetic patients have at least one chronic diabetic complication (hip fracture), all SARC-F patients will be defined as being at risk for diabetic sarcopenia.

The primary outcome was to determine whether type 2 diabetes had a higher rate of adverse events and in-hospital, short-, and long-term mortality. A subanalysis was conducted on diabetic and sarcopenic diabetic patients with hip fractures using the DS algorithm [18]. This algorithm was employed to classify patients into two groups: “possible sarcopenia” and “non-sarcopenia”. Subsequently, the primary statistical analysis was repeated within these two subgroups to explore differences and patterns specific to these classifications. Comorbidities other than diabetes and sarcopenia were controlled with the Charlson comorbidity Index. Sarcopenic diabetes related to one-year mortality was tested using a Cox proportional hazards model, which was defined according to the deaths observed during the 365 days following the incidence of hip fracture. In a second step, we conducted an adjusted Cox survival analysis, incorporating variables including sex, age, BI, CCI, and glycated hemoglobin.

### 2.4. Statistical Analysis

Data were collected retrospectively from the clinical records of admitted patients and analyzed using SPPS statistical software, version 23 (SPPS Inc., Chicago, IL, USA).

Absolute and relative frequencies (percentages) were used to describe qualitative variables (including dichotomous variables). Measures of central tendency (mean and median) and measures of dispersion (standard deviation, SD, and interquartile range, IQR) were used for quantitative variables. Bivariate analysis was performed for the variables included in the primary and secondary objectives, using Student’s *t*-test for quantitative variables with normal distribution and the Mann–Whitney U test for variables without normal distribution, and the chi-squared technique for qualitative variables. Multivariate analysis was performed using binary logistic regression for the main variable, calculating the crude and adjusted odds ratio (OR) for in-hospital mortality and adverse events. For the multivariate logistic regression models, the adjusting variables included age, the Charlson comorbidity index, and glycated hemoglobin, as these showed statistically significant differences in the bivariate analysis. Sex was also included based on clinical relevance despite not reaching significance in the univariate analysis. In addition to in-hospital mortality, we examined the association between sarcopenia and the occurrence of adverse events during hospitalization, including delirium, cardiac events, anemia, urinary tract infections (UTIs), and respiratory events. Odds ratios (ORs), 95% confidence intervals (CIs), and *p*-values were calculated both in unadjusted and adjusted models, incorporating the aforementioned covariates. For the analysis of mortality at 365 days, Cox regression was used to calculate the crude and adjusted hazard ratio (HR). Kaplan–Meier survival curves were then calculated. The significance level was set at *p* < 0.05.

### 2.5. Ethical Considerations

The study complied with regulatory requirements, good clinical practice guidelines, and the tenets of the Declaration of Helsinki (World Medical Association, October 2008 version on ethical principles for medical research in humans). As it was a retrospective study, informed consent was requested from patients when possible. In the rest of the cases, the anonymity of patients was ensured by separating the identifying data from the clinical data. The protocol was approved by the HULR Ethics and Clinical Research Committee (registration code HULR23122022, 12 January 2022).

## 3. Results

In total, 2631 patients were included in the study: 753 cases (28.6% of the sample) and 1878 controls. Cases were more than 1 year younger than controls and had higher comorbidities estimated by CCI and APR severity. The prevalence of chronic kidney disease was also higher in cases than in controls, with lower glomerular filtrate. The main baseline characteristics of the patients are shown in Table 1.

During hospitalization, cases had more intensive care unit (ICU) admissions, more complications (total and major complications), red blood cell transfusions, and in-hospital, 2-year, and 5-year mortality (Table 2).

Of the 753 diabetic patients hospitalized for hip fracture, 698 completed the SARC-F sarcopenia screening questionnaire and were subsequently evaluated for the presence of other specific DS risk factors, such as hip fracture, if the screening was positive. The prevalence of possible diabetic sarcopenia was 61.5% (429 patients). An analysis of the internal consistency of the data was first performed by correlating the presence or absence of possible sarcopenia with the Barthel Index, Lawton and Brody Index, Clinical Frailty Scale (CFS), Functional Ambulatory Category (FAC), and glycated hemoglobin levels. Except for glycated hemoglobin levels, which showed no significant difference, the presence of possible sarcopenia was strongly negatively correlated with the Barthel Index, Lawton and Brody Index, and FAC and positively correlated with the CFS (Table 3).

In the bivariate analysis, a lower age stood out in the group of patients with possible diabetic sarcopenia, together with a greater deterioration in basic and complex activities, a greater frailty estimated by the CFS, and a greater comorbidity estimated by the Charlson index. Among the comorbidities assessed in the sample, diabetic patients with possible sarcopenia had a higher prevalence of dementia and dysphagia (Table 4). This poorer functional and cognitive profile of patients with possible diabetic sarcopenia translated into longer hospital stay, higher overall number of complications, and poorer nutritional status estimated by the CONUT score at both hospital admission and discharge (Table 5).

In fact, patients with possible diabetic sarcopenia had a higher percentage of CONUT scores in the risk range for moderate and severe malnutrition on hospital admission and discharge. This poorer nutritional and functional status was associated with a higher incidence of urinary tract infection and delirium, and with higher short-, medium-, and long-term mortality: 30 days, 90 days, 180 days, and 1, 2, and 5 years (Table 5).

Despite the nutritional intervention during admission in these patients, which resulted in increased hospital prescription of enteral nutrition, no improvement in nutritional status at discharge was observed, nor did this translate into a reduction in the rate of complications or mortality.

On multivariate analysis, the presence of sarcopenia was associated with a higher risk of one-year mortality (odds ratio (OR) = 2.56, 95% confidence interval (95% CI) 1.84–3.57; *p* < 0.001). After adjustment for age and sex, possible sarcopenia estimated by SARC-F remained associated with the risk of death one year after hospital discharge (OR = 1.71 95%CI 1.23–2.37; *p* = 0.001); after adjustment for age, sex, and CCI score, the association remained significant (OR = 1.76 95%CI 1.27–2.44; *p* = 0.001); however, when glycated hemoglobin was added as an adjustment variable, the association lost significance (OR = 1.55 95%CI 0.69–3.51; *p* = 0.289).

**Figure 1:**
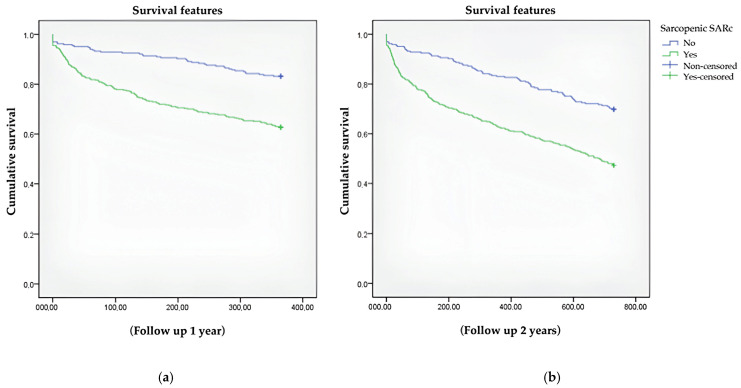
Analysis of survival at (**a**) 1 and (**b**) 2 years.

Analysis of survival at 1 and 2 years showed that patients with possible sarcopenia had a lower likelihood of survival at 1 (269.7 (SD 6.8) versus 328.8 (SD 5.8) days, *p* < 0.001) and 2 years (471.9 (SD 15.5) versus 607.7 (SD 13.7) days; *p* < 0.001) (Figure 1).

## 4. Discussion

Sarcopenic diabetes is a concept defining a new comorbidity of diabetes, characterized by significant muscle mass atrophy in individuals with diabetes mellitus (DM). Unlike common sarcopenia, it presents unique histological and physiological features specific to diabetic patients. The progressive loss of muscle mass and function is a hallmark of this condition, leading to an increased risk of falls, fractures, disability, and, ultimately, a significant reduction in quality of life [18].

The etiology of sarcopenic diabetes is linked to factors such as insulin resistance, poor glycemic control, chronic inflammation, and malnutrition. Insulin resistance reduces glucose uptake in muscles, impairing their function and strength. Furthermore, poor glycemic control (HbA1C ≥ 8.0%) is associated with significant muscle mass loss, while hyperglycemia promotes the accumulation of advanced glycation end-products (AGEs), which diminish muscle quality and strength. Chronic inflammatory states in DM, marked by elevated cytokines like TNF-α, contribute to muscle protein degradation. Additionally, some antidiabetic medications, such as sulfonylureas and SGLT2 inhibitors, may exacerbate muscle wasting [18]. Given the significant impact of sarcopenic diabetes on function and quality of life, structured screening and diagnosis are crucial in order to avoid further consequences [18].

In this real-world cohort analysis of patients who had suffered a major fracture, diabetic patients were younger compared to non-diabetic patients but had greater comorbidity burden, as reflected by a higher prevalence of CHF and chronic kidney disease, along with worse glomerular filtration rates. These factors translated into higher rates of intensive care unit admission, complications, blood transfusion, and overall blood cell unit requirements. Diabetic patients also showed higher in-hospital mortality, as well as higher 2-year and 5-year post-discharge mortality rates. Similarly, among diabetic patients, those with possible diabetic sarcopenia, as estimated by the diabetic sarcopenia algorithm [18], were older, had poorer functionality, a higher prevalence of dementia, and worse nutritional status, resulting in a longer length of stay, higher rate of complications, and higher mortality in the short, medium, and long term compared to diabetic patients without sarcopenia. In a multicenter study, both diabetes and cognitive impairment present on admission were associated with mortality at all-time intervals [16].

The prevalence of diabetes among patients with hip fracture was higher than in the general population. The same was true for the prevalence of sarcopenia in people with diabetes, which was higher than that estimated for people with hip fracture without diabetes and for people with diabetes without hip fracture. The prevalence of sarcopenia in hip fracture patients is reported to be between 44% [6] and 45.9% [24], compared with 12.9–40.4% in the general population [5]. However, a study conducted in Japan found a prevalence of sarcopenia in patients with hip fractures using the Asian Working Group for Sarcopenia (AWGS) criteria of 81.1% in men and 44.7% in women [25], and another study conducted in Spain using the European Working Group on Sarcopenia in Older People (EWGOSP) criteria reported a prevalence of 68.2% in men and 44.3% in women [26], highlighting the importance of managing sarcopenia in patients with hip fractures [27]. The lack of a significant association after adjusting for glycated hemoglobin suggests that glycemic control at the time of hospital admission does not influence the vital prognosis of diabetic patients with hip fracture and sarcopenia.

A retrospective cohort study found no association between sarcopenia and clinical outcomes during hospitalization [6]. A systematic review found a heterogeneous association between sarcopenia in hip fracture patients and postoperative mortality and length of stay [28]. However, our study found a statistical association between possible diabetic sarcopenia and length of hospital stay, in-hospital complications, and short- and long-term mortality. An association between sarcopenia, assessed by three different appendicular skeletal muscle (ASM) indices (37–61% in men and 11–26% in women), and long-term mortality has been described [6], but not specifically in diabetic patients.

As far as we know, this is the first trial to use the Diabetic Sarcopenia criteria with the classification algorithm specific to diabetic sarcopenia [18] in a real-world evidence cohort. In our sample, diabetic sarcopenia patients with hip fracture were frailer and had more comorbidities than non-sarcopenic diabetes patients. This worse clinical and functional profile could explain the higher mortality rate observed in sarcopenic diabetic patients, as also reported in a systematic review in which comorbidities (as estimated by the ASA score) and two emerging predictor factors—sarcopenia and frailty—were associated with mortality [29]. In our real-world evidence cohort, patients with sarcopenic diabetes had a higher proportion of cases classified as ASA grades II and III than hip fracture patients without sarcopenia. Nevertheless, sarcopenic diabetic patients in our study did not have lower serum vitamin D levels compared to diabetic patients without sarcopenia. This contrasts with findings from a previous study reporting a higher prevalence of vitamin D insufficiency and deficiency in sarcopenic patients [8].

Similarly, sarcopenic diabetic patients presented a longer length of stay, as reported in a paper where skeletal muscle index and paraspinal muscle density in hip fracture patients were associated with longer hospital stay and a higher number of blood transfusions during the perioperative period [30]. However, this latter finding has not been consistently replicated.

The presence of sarcopenic diabetes in hip fracture patients may increase the risk of worse clinical outcomes and mortality, as discussed throughout this manuscript. Sarcopenic diabetes is a new comorbidity factor associated with a higher prevalence of traditional comorbidities, frailty, and higher surgical risk as estimated by the ASA. This combination of risk factors in patients with serious clinical events such as hip fractures necessitates routine screening for diabetic sarcopenia. Identifying this condition can significantly improve the quality of life for postoperative hip fracture patients through accurate assessment for muscle regeneration and targeted rehabilitation after surgery [8].

Early detection to recover or maintain muscle mass in diabetic sarcopenic patients is crucial due to the significant impact that muscle loss has on overall health, functionality, and quality of life. Muscle tissue plays a crucial role in glucose metabolism, as it is responsible for a significant portion of insulin-mediated glucose uptake [18]. Increased muscle mass improves insulin sensitivity, facilitating better glycemic control, which is essential for managing diabetes and, as shown in our trial, directly impacts morbidity and mortality of diabetic patients.

This study had several limitations. First, the real-world data design allowed for the identification of associations but not causal relationships. In fact, sarcopenia in this study may be a consequence of the patients’ worse clinical profile rather than an etiological factor. Likewise, 8% of diabetic patients were lost to sarcopenia screening, and the total proportion of missing data was 12%, largely due to the retrospective nature of this study and the absence of some variable values in the clinical histories. Finally, a complete diagnosis of sarcopenia according to the EWGSOP2 (European Working Group on Sarcopenia in Older People) criteria was not feasible, as screening was performed solely with the SARC-F questionnaire, a tool that may have misclassified some patients. Another limitation was the lack of bone density data, as very few patients had undergone prior densitometry testing, precluding any meaningful analysis of this variable. Similarly, morpho-functional assessment was not conducted, which could have further aided in the accurate diagnosis of sarcopenia in elderly patients with hip fracture. Nevertheless, although the SARC-F is not a diagnostic instrument, the results and observed associations in our study are sufficiently robust to support its use in this context.

## 5. Conclusions

Diabetes and possible sarcopenic diabetes are common in older adults hospitalized for hip fracture. Likewise, both conditions increase the risk of worse outcomes and higher mortality in these patients. Routine screening for these conditions should be performed regularly in hip fracture patients to improve functional status and reduce the risk of adverse events. Also, further studies are needed to determine the best option to reverse or attenuate this condition during hospital admission for hip fracture.

## Figures and Tables

**Table 1:** Baseline characteristics of diabetic and non-diabetic patients.

Variable	Diabetes Group (n = 753)	Non-Diabetes Group (n = 1878)	*p*-Value
Sex, n (%)	♀ 530 (70.4%) ♂ 223 (29.6%)	♀ 1389 (74.0%) ♂ 489 (26.0%)	0.065
Age, years, mean (SD)	84.1 (SD 6.2)	85.3 (SD 6.4)	<0.001
HbA1c (SD)	6.7 (SD 1.1)	5.6 (0.7)	0.009
Charlson index, mean (SD)	3.8 (SD 2.5)	2.1 (SD 2.1)	<0.001
CONUT initial score, mean (SD)	3.0 (SD 1.9)	2.9 (SD 1.8)	0.125
CONUT risk of malnutrition, n (%)	No risk 152 (21.3%)	No risk 389 (21.8%)	0.301
	Mild risk 443 (62%)	Mild risk 1127 (63.1%)	
	Moderate risk 108 (15.1%)	Moderate risk 255 (14.3%)	
	Severe risk 12 (1.7%)	Severe risk 16 (0.9%)	
Glomerular filtration on hospital admission mL/min, mean (SD)	58.7 (SD 24.3)	65.1 (SD 23.9)	<0.001
All Patient Refined Diagnosis Related Groups (APR-DRGs), n (%)	I 291 (38.6%)	I 884 (47.1%)	<0.001
	II 379 (50.3%)	II 832 (44.3%)	
	III 76 (10.1%)	III 143 (7.6%)	
	IV 7 (0.9%)	IV 19 (1.0%)	
Dementia, n (%)	114 (15.1%)	288 (15.3%)	0.952
Chronic kidney disease, n (%)	113 (15.0%)	172 (9.2%)	<0.001
ASA n (%)	I 223 (29.6%)	I 714 (38.0%)	<0.001
	II 484 (64.3%)	II 1076 (57.3%)	
	III 46 (6.1%)	III 84 (4.5%)	
	IV 0 (0%)	IV 2 (0.1%)	
Vitamin D ng/mL, mean (SD)	14.7 (SD 17.2)	15.6 (SD 7.9)	0.634
Dysphagia, n (%)	10 (1.3%)	33 (1.8%)	0.107
CHF, n (%)	43 (5.7%)	117 (6.2%)	0.653
Chronic Obstructive Pulmonary Disease, n (%)	92 (12.1%)	202 (10.8%)	0.304
Stroke, n (%)	28 (3.7%)	44 (2.3%)	0.063

Note: American Society of Anesthesiology physical classification status system (ASA); Congestive Heart Failure (CHF). ♀ = male; ♂ = female.

**Table 2:** Differences in in-hospital outcomes between diabetic and non-diabetic patients.

Variable	Diabetes Group (n = 753)	Non-Diabetes Group (n = 1878)	*p*-Value
Length of stay, days, mean (SD)	8.3 (SD 5.5)	8.1 (SD 3.6)	0.374
CONUT score at hospital discharge, mean (SD)	3.0 (SD 2.2)	2.9 (SD 2.1%)	0.207
CONUT score, risk of malnutrition, n (%)	No risk 196 (27.4%)	No risk 481 (27.5%)	0.299
	Mild risk 359 (50.2%)	Mild risk 933 (52.2%)	
	Moderate risk 139 (19.4%)	Moderate risk 333 (18.6%)	
	Severe risk 21 (2.9%)	Severe risk 30 (1.7%)	
Glomerular filtration on hospital admission ml/min, mean (SD)	73.3 (SD 34.2)	92.8 (SD 38.7)	<0.001
Intensive care unit admission, n (%)	15 (2.0%)	15 (0.8%)	0.014
In-hospital mortality, n (%)	38 (5.0%)	63 (3.4%)	0.044
In-hospital complications, n (%)	413 (54.8%)	902 (48.0%)	0.002
In-hospital major complication, n (%)	404 (53.7%)	865 (46.1%)	<0.001
Number of in-hospital complications, mean (SD)	1.2 (SD 1.8)	0.9 (SD 1.3)	<0.001
Number of major in-hospital complications, mean (SD)	1.1 (SD 1.5)	0.8 (SD 1.1)	<0.001
Delirium, n (%)	71 (9.4%)	180 (9.6%)	0.942
Heart adverse event, n (%)	46 (6.1%)	111 (5.9%)	0.856
Anemia, n (%)	85 (11.3%)	184 (9.8%)	0.255
Urinary tract infection	35 (4.6%)	71 (3.8%)	0.324
Digestive adverse event, n (%)	7 (0.9%)	6 (0.3%)	0.061
Respiratory adverse event, n (%)	36 (4.8%)	94 (5.0%)	0.843
Surgical wound infection, n (%)	2 (0.3%)	12 (0.6%)	0.374
Respiratory infection, n (%)	10 (1.3%)	18 (1.0%)	0.405
Transfusion, n (%)	474 (62.9%)	1058 (56.3%)	0.002
Number of red blood cells units transfused, mean (SD)	1.7 (SD 1.9)	1.5 (SD 1.8)	0.004
Time to surgery, hours, mean (SD)	46.3 (SD 36.5)	45.4 (SD 30.2)	0.534
Time to surgery ≥ 48 h, n (%)	479 (63.6%)	1182 (62.9%)	0.755
Time to surgery ≥ 72 h, n (%)	121 (16.1%)	305 (16.2%)	0.953
In hospital mortality, n (%)	38 (5.0%)	63 (3.4%)	0.44
30-day mortality, n (%)	65 (8.6%)	163 (8.7%)	0.969
90-day mortality, n (%)	114 (15.1%)	262 (14.0%)	0.424
180-day mortality, n (%)	158 (21%)	358 (19.1%)	0.277
1-year mortality, n (%)	210 (27.9%)	473 (25.2%)	0.154
2-year mortality, n (%)	341 (45.3%	765 (40.7%)	0.036
5-year mortality, n (%)	418 (55.5%)	941 (50.1%)	0.012

**Table 3:** Correlations between SARC-F score and Barthel Index, Lawton and Brody Index, Clinical Frailty Scale, Functional Ambulatory Category, and glycated hemoglobin.

Variable	Coefficient	*p*-Value
Barthel	−0.80	<0.001
Lawton	−0.70	<0.001
CFS	0.85	<0.001
FAC	−0.84	<0.001
HbA1c	0.19	0.203

Note: Barthel: Barthel Index; Lawton: Lawton and Brody Index; CFS: Clinical Frailty Scale; HbA1c: Glycated hemoglobin.

**Table 4:** Baseline characteristics of diabetic patients with and without diabetic sarcopenia.

Variable	Sarcopenic Group (n = 429)	Non-Sarcopenic Group (n = 269)	*p*-Value
Sex n (%)	♀ 297 (69.2%) ♂ 132 (30.8%)	♀ 194 (72.1%) ♂ 75 (27.9%)	0.234
Age years, mean (SD)	84.5 (SD 5.7)	82.4 (SD 6.2)	<0.001
Barthel mean (SD)	49.0 (SD 23.7)	88.6 (SD 10.7)	<0.001
Lawton mean (SD)	0.7 (SD 1.22)	4.0 (SD 2.4)	<0.001
Clinical Frailty Scale mean (SD)	5.0 (SD 1.6)	1.8 (SD 1.0)	<0.001
HbA1c mean (SD)	7.5 (SD 1.8)	7.0 (SD 1.7)	0.133
Charlson index mean (SD)	3.8 (SD 2.3)	3.6 (SD 2.8)	0.203
Time to surgery, hours, mean (SD)	47.4 (SD 40.7)	42.9 (SD 27.3)	0.107
Hemoglobin level on hospital admission g/dL, mean (SD)	12.1 (SD 1.8)	12.2 (SD 1.7)	0.311
Glomerular filtration on hospital admission ml/min, mean (SD)	62.1 (SD 27.0)	58.6 (SD 22.6)	0.076
Vitamin D ng/mL, mean (SD)	14.2 (SD 12.2)	15.3 (SD 9.4)	0.330
CONUT score on hospital admission mean (SD)	3.1 (SD 1.9)	2.6 (SD 1.8)	0.005
CONUT score, risk of malnutrition, n (%)	No risk 67 (20.4%)	No risk 60 (28.7%)	0.072
	Mild risk 209 (63.5%)	Mild risk 127 (60.8%)	
	Moderate risk 46 (14.0%)	Moderate risk 20 (9.6%)	
	Severe risk 7 (2.1%)	Severe risk 2 (1.0%)	
Dysphagia n (%)	111 (25.9%)	12 (4.5%)	<0.001
Chronic heart disease n (%)	27 (6.3%)	15 (5.6%)	0.746
Chronic Obstructive Pulmonary Disease n (%)	60 (14.0%)	27 (10.0%)	0.128
Stroke n (%)	21 (4.9%)	3 (1.1%)	0.009
Dementia n (%)	97 (22.6%)	14 (5.2%)	<0.001
Chronic kidney disease n (%)	62 (14.5%)	48 (17.8%)	0.242

♀ = male; ♂ = female.

**Table 5:** Differences in in-hospital results between sarcopenic and non-sarcopenic groups.

Variable	Sarcopenic Group (n = 429)	Non-Sarcopenic Group (n = 269)	*p*-Value
Length of stay, days, mean (SD)	8.4 (SD 3.7)	7.7 (SD 2.7)	0.018
Intensive care unit admission n (%)	5 (1.2%)	8 (3.0%)	0.147
Number of complications mean (SD)	1.3 (SD 1.6)	1.1 (SD 1.3)	0.048
Number of major complications mean (SD)	1.2 (SD 1.5)	1.0 (SD 1.2)	0.051
Number of red blood cells units transfused, mean (SD)	1.8 (SD 1.7)	1.6 (SD 1.7)	0.436
Hemoglobin at hospital discharge g/dL, mean (SD)	10.4 (SD 1.2)	10.3 (SD 1.1%)	0.102
Glomerular filtrate at hospital discharge ml/min mean (SD)	78.1 (SD 39.8)	72.8 (SD 33.1)	0.066
CONUT score, risk of malnutrition, n (%)	No risk 54 (13.2%)	No risk 56 (21.5%)	0.496
	Mild risk 215 (52.9%)	Mild risk 136 (52.3%)	
	Moderate risk 77 (19.0%)	Moderate risk 35 (13.5%)	
	Severe risk 61 (14.9%)	Severe risk 33 (12.7%)	
Barthel index	49.6 (25.0)	56.3(26.2)	0.205
CONUT score at hospital discharge mean (SD)	3.1 (SD 2.3)	2.4 (SD 2.2)	0.001
Delirium n (%)	58 (13.5%)	14 (5.2%)	<0.001
Acute heart adverse event n (%)	27 (6.3%)	9 (3.3%)	0.113
Anemia during hospital admission n (%)	50 (11.7%)	32 (11.9%)	1.000
Urinary tract infection n (%)	23 (6.1%)	7 (2.6%)	0.043
Acute digestive adverse event n (%)	5 (1.2%)	1 (0.4%)	0.414
Acute respiratory adverse event n (%)	24 (5.6%)	7 (2.6%)	0.088
Surgical wound infection n (%)	2 (0.5%)	0 (0%)	0.526
Respiratory infection n (%)	7 (1.6%)	3 (1.1%)	0.748
Transfusion n (%)	284 (66.2%)	166 (61.7%)	0.130
Time to surgery, hours mean (SD)	40.8 (SD 2.0)	27.3 (SD 1.7)	0.078
Time to surgery ≥ 48 h n (%)	164 (38.2%)	84 (31.2%)	0.062
Time to surgery ≥ 72 h n (%)	76 (17.7%)	34 (12.6%)	0.087
In-hospital mortality n (%)	22 (5.1%)	9 (3.3%)	0.346
30-day mortality n (%)	59 (13.8%)	13 (4.8%)	<0.001
90-day mortality n (%)	92 (21.4%)	21 (7.8%)	<0.001
180-day mortality n (%)	124 (28.9%)	27 (10.0%)	<0.001
1-year mortality n (%)	163 (38%)	47 (17.5%)	<0.001
2-year mortality n (%)	227 (52.9%)	82 (30.5%)	<0.001
5-year mortality n (%)	324 (75.5%)	126 (46.8%)	<0.001

## Data Availability

The original contributions presented in this study are included in the article. Further inquiries can be directed to the corresponding author, due to ethical reasons.
